# A Transcriptome Analysis Revealing the New Insight of Green Light on Tomato Plant Growth and Drought Stress Tolerance

**DOI:** 10.3389/fpls.2021.649283

**Published:** 2021-10-21

**Authors:** Zhonghua Bian, Yu Wang, Xiaoyan Zhang, Steven Grundy, Katherine Hardy, Qichang Yang, Chungui Lu

**Affiliations:** ^1^Photobiology Research Center, The Institute of Urban Agriculture, Chinese Academy of Agricultural Sciences, Chengdu, China; ^2^School of Animal, Rural and Environment Sciences, Nottingham Trent University, Brackenhurst Campus, Nottingham, United Kingdom; ^3^Institute of Industrial Crops, Jiangsu Academy of Agricultural Sciences, Nanjing, China

**Keywords:** green light, stomatal aperture, ABA, drought stress, transcriptome, tomato

## Abstract

Light plays a pivotal role in plant growth, development, and stress responses. Green light has been reported to enhance plant drought tolerance via stomatal regulation. However, the mechanisms of green light-induced drought tolerance in plants remain elusive. To uncover those mechanisms, we investigated the molecular responses of tomato plants under monochromatic red, blue, and green light spectrum with drought and well-water conditions using a comparative transcriptomic approach. The results showed that compared with monochromatic red and blue light treated plants, green light alleviated the drought-induced inhibition of plant growth and photosynthetic capacity, and induced lower stomatal aperture and higher ABA accumulation in tomato leaves after 9 days of drought stress. A total of 3,850 differentially expressed genes (DEGs) was identified in tomato leaves through pairwise comparisons. Functional annotations revealed that those DEGs responses to green light under drought stress were enriched in plant hormone signal transduction, phototransduction, and calcium signaling pathway. The DEGs involved in ABA synthesis and ABA signal transduction both participated in the green light-induced drought tolerance of tomato plants. Compared with ABA signal transduction, more DEGs related to ABA synthesis were detected under different light spectral treatments. The bZIP transcription factor- HY5 was found to play a vital role in green light-induced drought responses. Furthermore, other transcription factors, including WRKY46 and WRKY81 might participate in the regulation of stomatal aperture and ABA accumulation under green light. Taken together, the results of this study might expand our understanding of green light-modulated tomato drought tolerance via regulating ABA accumulation and stomatal aperture.

## Introduction

In nature, plants often suffer from adverse environmental conditions, including drought, extreme temperature, heavy metal, and salinity stress. To survive under different stress conditions, plants must optimize their growth and development at the expense of yield loss. Drought is one of the most important prevalent abiotic stresses that limits crop growth and productivity (Somerville, 2001; Lesk et al., 2016). With the global temperature increasing and worldwide population growth, the scarcity of water resources in agriculture will aggravate crop loss. The traditional producing staple foods could not meet the future food requirement due to the shortage of sufficiency irrigation water, and drought and/or unstable rainfall. Thus, new strategies for improving crop drought tolerance and water use efficiency are urgently needed for the next generation of agriculture (Chen et al., 2020).

Vertical Farming has been branded as the future of Food Production due to the environmental benefits and food security benefits (small geographical footprint, pesticide-free, water reuse, all year round growing) (Kalantari et al., 2020). However, there are still some issues that limit the wide commercialization of vertical farming, such as high energy cost, limited crop choice and lack of specialized crop varieties (Kozai and Niu, 2015). LEDs offer cheap, cool, controllable sources of light that can selectively and quantitatively provide different wavelengths that can activate discrete developmental pathways to change leaf area, thickness, stem length through photoreceptors include phytochrome and cryptochrome (Kozai and Niu, 2015). This provides us with a new opportunity to manipulate the quality and quantity of produce for markets and meet the demands of retailers. In order to understand the molecular mechanism leading to an increase in the resource use efficiency (light, water and nutrients). It is necessary to identify key genes (via transcriptomic analysis) which act as molecular markers and regulators for vertical framing (indoor farming) crop breeding.

It is reported that 70% of global freshwater is used for agriculture (Döll, 2009; FAO, 2012). Most of the water used in agriculture is lost to the atmosphere by evaporation and transpiration that is regulated by stomatal movement. Stomata are the important epidermal leaf pores in response to the water states of plants. It is evident that the early response of stomatal movement to drought stress can help plant survival sometimes through maintaining high relative water content (RWC) in leaves (Reddy et al., 2004). The plant hormone abscisic acid (ABA) is the key regulator of stomatal movement, which plays a pivotal role in plant's adaptive response to drought stress (Nakashima and Yamaguchi-Shinozaki, 2013). The ABA in plants is indirectly synthesized through the carotenoid pathway using β-carotene as a precursor (Schwartz and Zeevaart, 2010). The 9-cis-epoxycarotenoid dioxygenase (NCED) encoded by the homologous genes of *VIVIPAROUS14* (*VP14*), converses of 9′-cis-neoxanthin and 9′-cis-violaxanthin into xanthoxin in the plastid of plant cells. In the cytoplasm, the resulting xanthoxin will be further converted into two crucial enzymes: ABSCISIC ACID 2 (ABA2) and *Arabidopsis* aldehyde oxidase 3 (AAO3) (Chen et al., 2020). NCEDs are the rate-limiting enzymes of ABA synthesis. Overexpression of NCED related genes has been proved to enhance plant drought tolerance via increasing ABA levels to trigger stomatal closure and reduce transpiration (Wan et al., 2009; Lee and Luan, 2012). However, the decrease of stomatal aperture or stomatal closure caused by drought stress usually leads to a decrease in photosynthesis and finally results in a relatively low yield (Mafakheri et al., 2010). Thus, it is important to increase plant drought tolerance and concomitantly stabilize photosynthesis to minimize the drought-induced yield losses when carrying out stomatal regulation in crop production. Given the importance of endogenous ABA in regulating stomatal response to drought stress and the complexity of genetic engineering approaches used in enhancing plant drought stress tolerance, economic and innovative approaches are urgently needed for improving crop drought tolerance.

Light not only provides energy for driving photosynthesis but also works as a signal to regulate plant growth, development, and stress responses in a phytochrome-dependent manner (Wang et al., 2018). Light and ABA are integrated at the molecular level to regulate seed germination and seedling development. LONG HYPOCOTYL 5 (HY5, a bZIP transcription factor) plays an important role in integrating light signals with endogenous ABA pathways to help plants better adapt to environmental stresses (Chen et al., 2008; Xu et al., 2014). Recently, increasing numbers of studies demonstrate that endogenous ABA metabolism and/or ABA signaling pathway are subjected to the regulation of light spectra, including blue, red, and far-red light (Wang et al., 2018; Stawska and Oracz, 2019). In our previous studies, we found that green light enhanced tomato drought tolerance via altering stomatal aperture and ABA-dependent transcription factor-AREB1 (Bian et al., 2019). However, how green light induces ABA signals to regulate plant drought tolerance at the molecular level remains largely unclear.

Ribonucleic acid sequencing (RNA-seq) analysis based on next-generation sequencing is one of the main approaches of bioinformatics. RNA-seq is a good method for whole-transcriptome investigation (Cao et al., 2016). In this study, the molecular mechanism pathways and key genes of drought-treated tomato seedling response to green light were identified using RNA-seq analysis. Based on plant physiological responses and analyses of the Kyoto Encyclopedia of Genes and Genomes (KEGG) and Gene Ontology (GO) enrichment, a model of green light-enhanced tomato drought tolerance was proposed. Our present study could not only further facilitate our understanding of light spectra-regulated drought tolerance at the genome-wide level but also could identify key regulators and genes for improving stress tolerance of tomato grown under controlled environments.

## Materials and Methods

### Plant Materials and Growth Conditions

Seeds of tomato (*Solanum Lycopersicum* L. cv. Ailsa Craig; wild type) were soaked in distilled water for 8 h and then grown in dark for 48 h. These germinated seeds were sown in rock wool cubes (3 × 3 × 4 cm^3^) and grown under white LED light (Heliospectra RX30, Sweden) as our previous study (Bian et al., 2019) with photosynthetic photon flux density (PPFD) and photoperiod at 200 μmol m^−2^s^−1^ and 16 h, respectively, in an environmentally controlled growth chamber. The day/night temperature, air relative humidity, and CO_2_ level were 25/20°C, 65%, and 400 μmol mol^−1^, respectively. These seedlings were watered with half-strength Hoagland nutrition solution every other day.

### Drought and Light Treatments

After around 28 days, similarly sized and healthy seedlings with five true leaves were transplanted into rock wool media (7.5 × 7.5 × 6.5 cm^3^). The rock wool media were watered using half-strength Hoagland solution to reach their full water-holding capacity before these seedlings were transplanted. Throughout this whole experiment, these tomato seedlings were randomly grown under two watering regimes (well-watered and drought-stressed conditions) and concomitantly exposed to different light spectra. The two watering regimes: (1) well-watered, 90 ± 5% water-holding capacity of rock wools and (2) drought, stressed, non-watered until the plants showed severe drought stress symptoms–obvious turgor loss and wilting. The moisture of rock wools was monitored using every other day in proximity of the roots with a portable HH2 Moisture Meter connected to a WET sensor (Delta-T Device LTD, Cambridge, UK). The irrigation strategy was performed as the method described in the previous study (Wang et al., 2013; Bian et al., 2019). The light treatments included monochromatic red (peak at 660 nm), blue (peak at 450 nm) and green (peak at 530 nm) LED light (Heliospectra RX30, Sweden). The three different light treatments were combined with well-watered or drought conditions. In the first three treatments, plants were grown under well-watered conditions and exposed to monochromatic red light (RW), blue light (BW), or green light (GW) with PPFD at 200 μmol m^−2^ s^−1^. In the other three were drought treatments. These drought-treated plants were irradiated with 200 μmol m^−2^ s^−1^ monochromatic red light (RD), blue light (BD), or green light (GD), respectively.

### Plant Growth and Abscisic Acid Determination

After being treated for 9 days, the second fully expanded leaves from the top of plants were collected and immediately frozen in liquid nitrogen before being stored at −80°C. The extraction and determination of abscisic acid (ABA) were carried out as described by Balcke et al. (2012). Furthermore, another eight plants were randomly selected for the measurements of plant height, biomass, and leaf area measurements as the method of Bian et al. (2019). The leaf area was determined using a leaf area meter (LI-3000C, LI-COR, NE, USA).

### Gas Exchange and Stomatal Aperture Determination

The second youngest and fully expanded leaves of plants under different treatments were used for gas exchange determination before (Day 0) and after treatment (Day 3, 6, and 9). The net photosynthesis (A_net_) and chlorophyll fluorescent parameters were concomitantly measured using a portable photosynthesis system (LI-6800 F, LI-COR, Inc., Lincoln, NE). The light response curve of A_net_ was measured as the protocol of LI-6800F, and the light intensities were set as following: 0, 30, 50, 100, 200, 500, 800, and 1,200 μmol m^−2^ s^−1^. The data obtained at the PPFD of 200 μmol m^−2^s^−1^ were used to analyze the photosynthetic performance of plants. During the gas exchange measurement, actinic light in the leaf chamber was provided by red and blue LED light sources (90% red, 10% blue), while the CO_2_ level, air temperature, and airflow were set at 400 μmol mol^−1^, 25^o^C, and 500 μmol s^−1^, respectively. The light response curve fitting was carried out according to the methods of Thornley (1976). The responses of photosystem II (PSII) quantum efficiency (Φ_PSII_) to the changes of PPFD were calculated as described by Baker (2008). The length and width of the stomata were determined using the method of Zeng et al. (2008). The stomatal aperture was calculated as the ratio of stomatal width to length.

### Relative Water Content and Cell Membrane Stability

The method of Pan et al. (2012) was used to determine the relative water content (RWC) of plants treated with different light and water conditions. The cell membrane stability was expressed as the electrolyte leakage. The electrolyte leakage was determined as described by Jungklang et al. (2017). Briefly, five leaf discs punched from the second youngest and fully expanded leaves were put into a test tube with 20 ml of distal water and shaken every 5 min. After 30 min, the conductivity was measured using a conductivity meter. The total conductivity was measured after the test tubes were boiled for 15 min. The electrolyte leakage was calculated as the percentage of total conductivity.

### RNA Extraction and Transcriptome Sequencing

After 9 days of treatments, the second fully expanded leaves from the top of randomly selected 12 plants (four plants per sample, three samples per treatment) were collected for RNA extraction. The total RNAs for transcriptome sequencing were extracted using an RNeasy Plant Mini RNA isolation kit (Qiagen, Hilden, Germany) according to the manufacturer's instructions. The quantity and purification of total RNAs were determined using a Nanodrop 2000C spectrophotometer (Thermo Scientific, USA) before and after the total RNAs were treated with 50 μl of RNase-free DNase I (Sigma-Aldrich, Poole, UK) at 37°C for 15 min. The integrity of total RNAs was rechecked using the Agilent Bioanalyzer 2100 system (Agilent Technologies, USA).

The total RNA extracted from each leaf sample was used for RNA-Seq library construction and sequencing by Biomics (Beijing) Biotech Co, Ltd. For the RNA sample preparations, 3 μg of RNA per sample was used as input material. The NEBNext® UltraTM RNA Library Prep Kit for Illumina® (NEB, USA) was used for generating sequencing as the manufacturer's instructions. Briefly, mRNA was purified from total RNA using poly-T oligo-attached magnetic beads. Fragmentation was carried out using divalent cations under elevated temperature in NEBNext First Strand Synthesis Reaction Buffer (5X). First-strand cDNA was synthesized using random hexamer primer and M-MuLV Reverse transcriptase (RNase H-), while the second strand cDNA was synthesized using DNA Polymerase I and RNase H. Remaining overhangs were converted into blunt ends via exonuclease/polymerase activities. The library fragments were purified with AMPure P system (Beckman Coulter, Beverly, USA) to select cDNA fragments of referentially 250~300 bp in length. Then PCR was performed with Phusion High -Fidelity DNA polymerase, Universal PCR primers, and Index (X) Primer. Finally, the enriched cDNA libraries were assessed using the Agilent Bioanalyzer 2100 system before being sequenced on the HiSeq 6000 sequencing platform (Illumina, USA) to generate 125/150 bp paired-end reads.

### Transcriptomic Analysis

The raw reads were cleaned by discarding the reads with adaptor contamination and low-quality reads (a quality score of *Q* <20). Clean reads from individual libraries of each group were mapped to the tomato reference genome Hisat2 v2.0.5 (Kim et al., 2015). The gene expression levels were estimated by the FPKM (fragments per kilobase of per millions of fragments mapped) of each gene calculated based on the length of the reads count mapped gene using RSEM (RNA-Seq by Expectation Maximization) module provided within the Trinity package (Trapnell et al., 2009). The DESeq2 R package (1.16.1) was used to carry out differential expression analysis of pairs of treatments (three biological replicates). The Benjamini and Hochberg's approach was used to adjust the resulting *p*-values for controlling the false discovery rate. Genes with an adjusted *p*-value < 0.05 and |log_2_ Fold Change| <1 found by DESeq2 were assigned as differentially expressed between pairs of treatments. The analysis of the Gene Ontology (GO) and Kyoto Encyclopedia of Genes and Genomes (KEGG) pathways enrichment of differential expression genes (DEGs) were performed using the cluster Profiler R package was used to carry out (Tarazona et al., 2011). The GO and KEGG terms with corrected *p* < 0.5 were considered significantly enriched by DEGs between the two treatments.

### Protein-protein Interaction Network and TF Regulatory Analysis

The PPI pairs of DEGs were extracted from STRING version 10.5 (Damian et al., 2017). Cytoscape version 3.8.0 was used to establish the PPI network of screened DEG (Shannon et al., 2003). The top five hub DEGs involved in the PPI network were identified from the network based on radiality by employing cytohubba (Chaudhary et al., 2019).

### Validation of RNA-Seq Data by qRT-PCR Analysis

Ten DEGs were randomly selected for the validation of RNA-seq results by qRT-PCR. A total amount of 1 μg RNA, which was the same as that used for the RNA-seq sequence, was used for cDNA synthesis was performed through a RevertAid First Strand cDNA Synthesis Kit (Thermo Scientific, USA). The primers for these selected genes were designed by Primer Premier 6.0. Tomato Actin gene was employed as an internal reference gene (Li et al., 2016). The sequences of these primers are summarized in Supplementary Table 1. The qRT-PCR was performed on a CFX Connect™ Real-Time PCR Detection System (Bio-Rad, Hercules, CA, USA) with SsoFast™ EvaGreen® Supermix (Bio-Rad). The thermocycling conditions were set to 95°C for 30 s and 40 cycles of 95 C for 5 s, 56°C for 5 s, and 60°C for 5 s, followed by a melting curve (65–95°C). The qPCR was performed in triplicate, with three total RNA samples extracted from nine plants (three plants per sample). The relative gene expression levels of these selected genes were calculated using the 2^−Δ*ΔCt*^ method (Shannon et al., 2003).

## Results

### The Plant Growth of Tomato Exposed to Various Light Spectra Under Different Water Conditions

The growth of tomato seedlings was significantly affected by light spectra under both well-water and drought conditions (Table 1). Under the well-water condition, the plant height was highest under RW, followed by GW and then BW. The leaf area and plant dry weight of GW were lower than those under RW and BW. Except for the plant height and leaf dry weight, no significant differences were observed in those studied parameters between RW and BW. Drought led to significant decreases in plant height and dry weight of plants grown under red and blue light but showed slightly negative effects on those parameters of plants exposed to green light. Furthermore, the leaf area and dry weight of plants grown under RD, BD, and GD were comparable to each other. The leaf areas of RD, BD, and GD decreased by 31.61, 38.20, and 10.02%, while the total dry mass decreased by 32.03, 26.16, and 7.79% when compared with those parameters of RW, BW, and GW, respectively. These results indicate that green light counteracted the negative effects of drought stress on the tomato seedling growth.

**Table 1:** The growth of tomato seedlings under different water and light spectral conditions (*n* = 6–8).

**Treatments**	**Plant height (cm)**	**Leaf area (cm^**2**^)**	**Dry weight (g)**		
			**Leaf**	**Stem**	**Leaf + stem**
RW	27.23 ± 0.85 a	323.75 ± 18.07 a	0.53 ± 0.08 b	0.50 ± 0.05 a	1.03 ± 0.10 a
BW	24.74 ± 1.19 b	331.47 ± 39.76 ab	0.66 ± 0.03 a	0.48 ± 0.05 a	1.15 ± 0.08 a
GW	25.05 ± 1.02 b	272.44 ± 17.33 b	0.45 ± 0.09 b	0.32 ± 0.03 c	0.77 ± 0.12 b
RD	24.68 ± 1.39 b	221.36 ± 27.34 c	0.37 ± 0.06 b	0.33 ± 0.06 bc	0.70 ± 0.05 b
BD	21.84 ± 0.89 c	204.87 ± 16.48 c	0.43 ± 0.06 b	0.41 ± 0.03 b	0.84 ± 0.09 b
GD	24.81 ± 1.49 b	199.16 ± 29.01 c	0.37 ± 0.08 b	0.35 ± 0.03 bc	0.71 ± 0.12 b

*The data are presented as Mean ± SEs. The different letters in each column indicate significant differences (p < 0.05) among treatments*.

### The Photosynthetic Performance of Tomato Plants Exposed to Different Light Spectra

**Figure 1:**
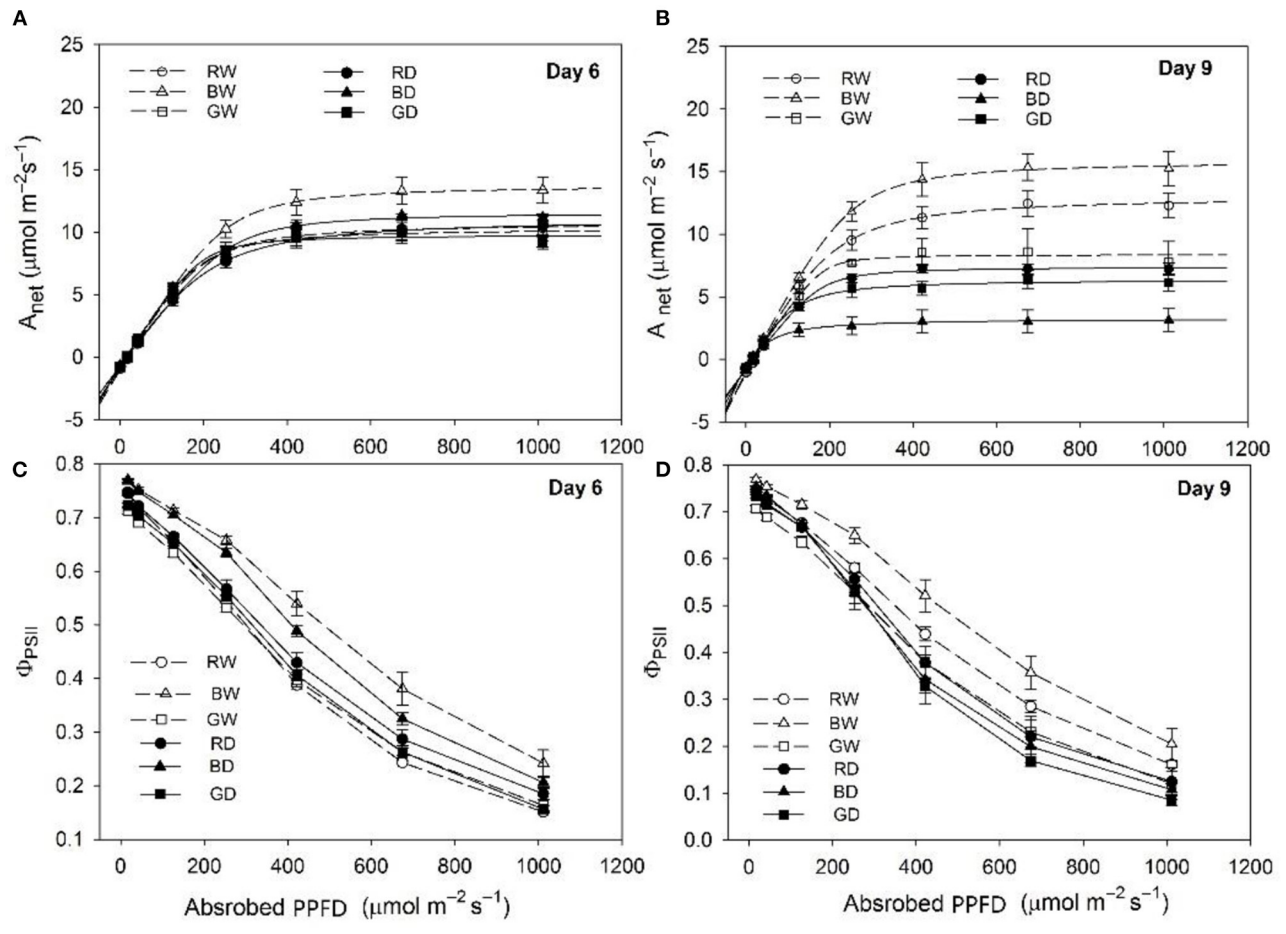
The photosynthetic response of tomato seedlings under different water and spectral conditions. **(A,B)**, the changes of net photosynthetic rate (A_net_) to increased light intensity; **(C,D)**, the changes of photosystem II (PSII) quantum efficiency (ϕ_PSII_). RW, BW, and GW: well-water combined with red, blue, and green LED light, respectively; RD, BD, and GD: drought stress combined with red, blue, and green LED light, respectively. The photosynthetic photon flux density (PPFD) for all the treatments was 200 μmol m^−2^ s^−1^.

Drought stress led to marked decreases in max A_net_ and Φ_PSII_ of tomato plants under red and blue light. However, the levels of those parameters were comparable between GW and GD (Figure 1). Under well-watered conditions, the max A_net_ was highest under BW, flowed by RW and then GW throughout this study (Figures 1A,B). The changes of A_net_ with the increasing of light intensities (A-Q curve) were comparable among BD, RD, and GD at day 6 (Figure 1A), while the A-Q curve of plants under BD was markedly lower than those under RD and BD at day 9 (Figure 1B). Furthermore, Φ_PSII_ values of plants under different treatments decreased with the increase of light intensity. The values of Φ_PSII_ under BW and BD were higher than those under the other four treatments, while the Φ_PSII_ did not differ significantly among RW, GW, RD, and GW at day 6 (Figure 1C). The highest values of Φ_PSII_ were also observed under BW, followed by RW and GW, while values of this parameter under BD, RD, and GD were comparable to that under GW at day 9 (Figure 1D).

### The Stomatal Responses and ABA Content of Tomato Plants Under Different Light Spectra

**Figure 2:**
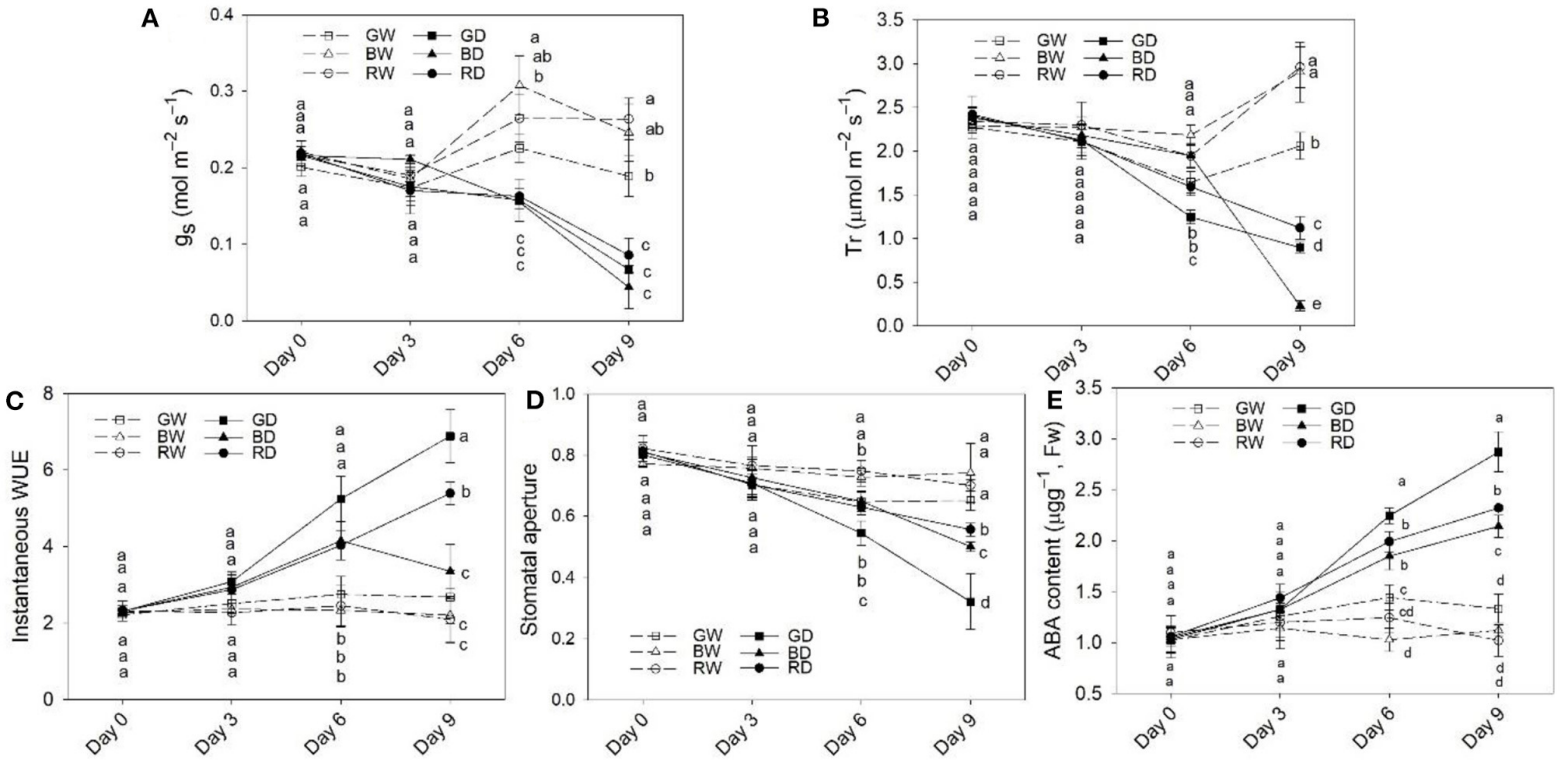
The stomatal response, water use efficiency (WUE), and abscisic acid (ABA) content in leaves of plants under different water and light spectra condition. **(A,B)**, The responses of stomatal conductance (g_s_) and transpiration rate (Tr) of plant leaves; **(C)**, The instantaneous WUE; **(D)**, stomatal aperture; **(E)**, ABA content in plant leaves after 9 days of treatment. RW, BW, and GW: well-water combined with red, blue, and green LED light, respectively; RD, BD, and GD: drought stress combined with red, blue, and green LED light, respectively. The PPFD for all the treatments was 200 μmol m^−2^ s^−1^.

The g_s_ and Tr were markedly decreased between Day 6 and Day 9 (Figures 2A,B). The g_s_ showed a similar change tendency during the 9 days of treatments, but the g_s_ of plants under GD was lower than that under BD and RD (Figure 2A). Unlike g_s_, Tr was significantly affected by light spectra. Under the well-watered condition, the Tr levels of plant leaves under GW were lower than those under RW and BW on day 6 and day 9. After 6 days of drought treatment, the Tr differed significantly among drought treatments, with the highest values observed under BD, followed by RD, and then by GD, while Tr under BD was markedly lower than those under RD and GD on day 9 (Figure 2B). Drought stress led to increases in instantaneous WUE. The values of instantaneous WUE differed significantly among GD, BD, and RD with the highest value detected under GD, followed by RD and then BD (Figure 2C) at Day 9. Under the well-water condition, there were no marked differences in instantaneous WUE among different light spectra. Regardless of water conditions, the stomatal apertures of green light treated plants were lower than those under red and blue light between Day 6 and Day 9 (Figure 2D).

To verify the involvement of ABA in the response of plants to light spectra, the ABA content in tomato leaves of different treatments was determined. Under well-water conditions, the ABA content was comparable to each other, but the level of ABA under GW was slightly higher than that under BW and RW. However, under drought conditions, the ABA content was significantly affected by light spectra. The ABA content was highest under GD, followed by RD, while the lowest value was observed under BD from Day 6 to Day 9 (Figure 2E).

### The Responses of Water Status and Plant Phenotype of Tomato Exposed Different Light Spectra

**Figure 3:**
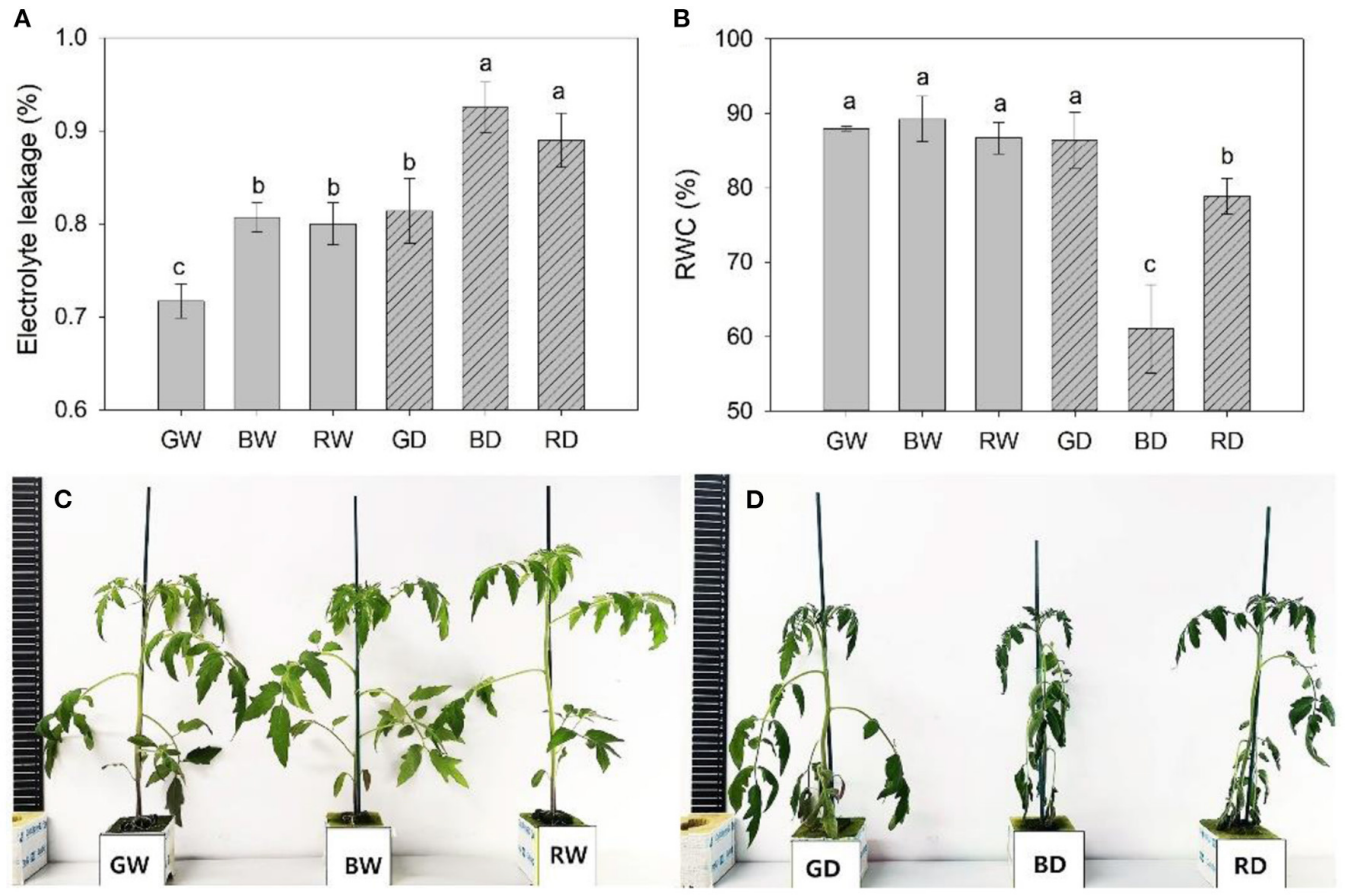
The electrolyte leakage **(A)**, relative water content [RWC, **(B)**], and phenotype **(C,D)** of plants were treated with different water and light spectral condition for 9 days. RW, BW, and GW: well-water combined with red, blue, and green LED light, respectively; RD, BD, and GD: drought stress combined with red, blue, and green LED light, respectively. The PPFD for all the treatments was 200 μmol m^−2^ s^−1^.

Drought for 9 days led to a marked increase in electrolyte leakage. The lowest electrolyte leakage was observed in green light treated plants under both well-watered and drought conditions. However, there was no significant difference in electrolyte leakage between red and blue light, as shown by the comparable values of this parameter between RW and BW, and between RD and DW (Figure 3A). When compared with the other four treatments, the RWC of BD and RD markedly decreased with the lowest value observed under BD. It is worth noting that no significant difference was observed in RWC between GW and GD (Figure 3B). These results indicate green light shows a positive function on enhancing the plant drought tolerance, as being further validated by the different wilting phenotypes of plants irradiated red, blue, green light under drought stress (Figures 3C,D).

### Identification of DEGs of Tomato Seedlings Under Different Light Spectra by RNA-Seq

**Figure 4:**
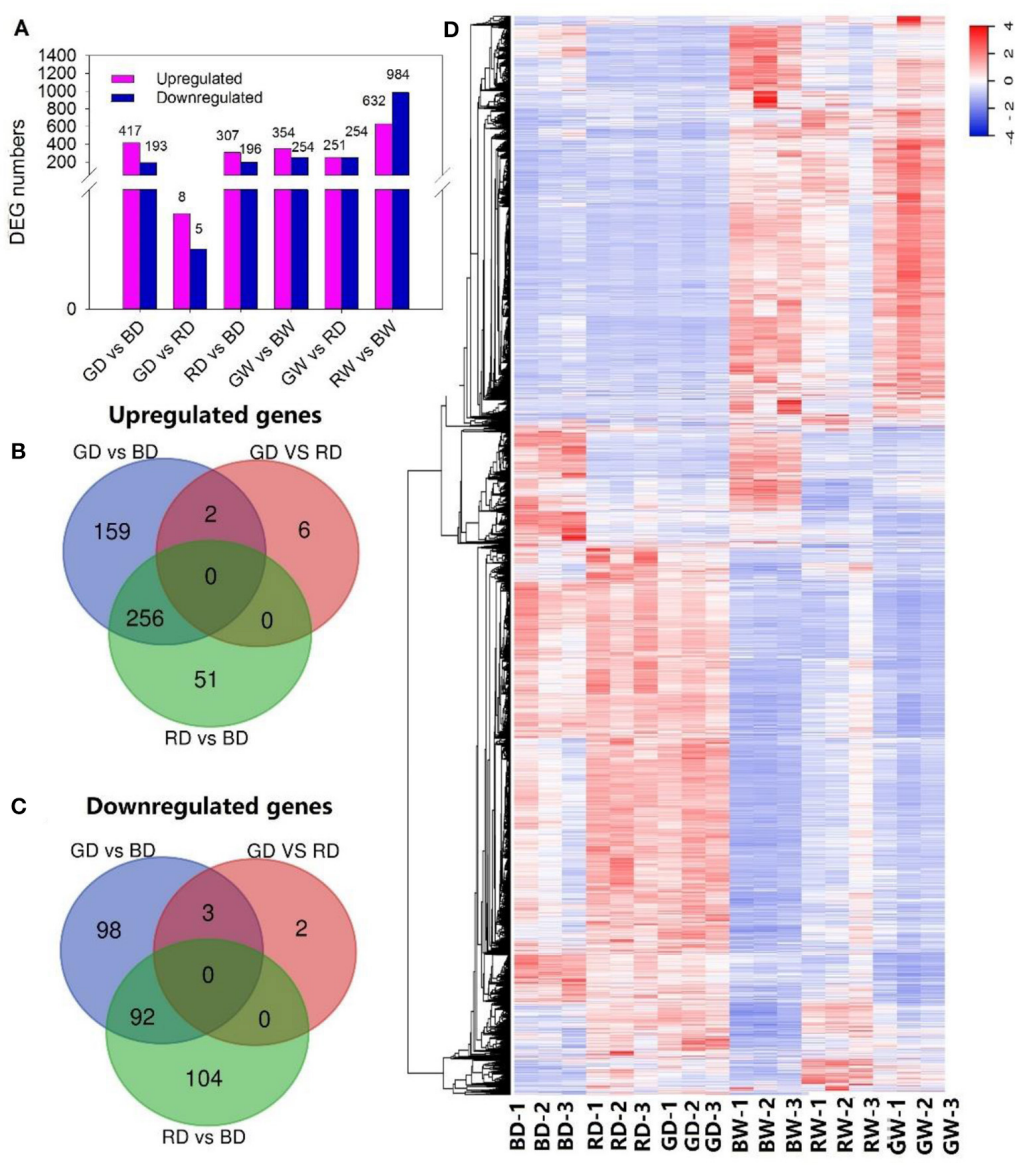
The changes in gene expression profiles of tomato treated with different light spectra and water conditions. **(A)**, The number of DEGs between treatments; **(B,C)**, Venn diagram presenting up-regulated and down-regulated genes among plants exposed to different light spectra under drought conditions; **(D)**, Hierarchical clustering of DEGs, fragments per kilobase of transcript per million fragments mapped (FPKM), and relative expression of DEGs between different light and water treatments were calculated as log_2_ (FPKM) of differentially expressed genes, log_2_ (FPKM) of differentially expressed genes were calculated. A scale indicating the color assigned to log_2_ (FPKM) is shown to the right of the cluster.

To further investigate key genes involved in the regulation of tomato seedling drought tolerance under different light spectra, we performed transcriptome analysis of plant leaves under red, blue, and green monochromatic light after 9 days of drought and well-watered treatment. A total of 3,850 DEGs was identified through pairwise comparisons. There were 601, 13, and 503 DEGs in the comparisons of GD vs. BD, GD vs. RD, and RD vs. BD, while 608, 505, and 1,616 DEGs were recorded when comparing GW vs. BW, GW vs. RW, and RW vs. BW (Figure 4A). Especially, 417, 8, and 307 DEGs were up-regulated, whereas 183, 5, and 196 DEGs were down-regulated in the comparison of GD vs. BD, GD vs. RD, and RD vs. BD, respectively. A total of 159, 6, and 51 DEGs were up-regulated uniquely (Figure 4B), while the number of uniquely down-regulated DEGs were 98, 2, and 104 between GD and BD, GD and RD, and RD and BD, respectively (Figure 4C).

In addition, a hierarchical clustering analysis was performed to present a general overview of the expression pattern of DEGs (Figure 4D). Most of the genes with higher expression levels under drought stress displayed lower transcription levels under well-watered conditions and vice versa. Furthermore, the expression profile of most genes under blue light showed a great difference when compared with those of red and green light treated leaves, especially under drought stress. Notably, most DEGs presented different transcription profits between red and green light under well-watered conditions; whereas most identified genes showed a similar transcription pattern under drought stress (Figure 4D).

### Functional Classification of DEGs Responses to Different Light Spectra Under Drought Stress

**Figure 5:**
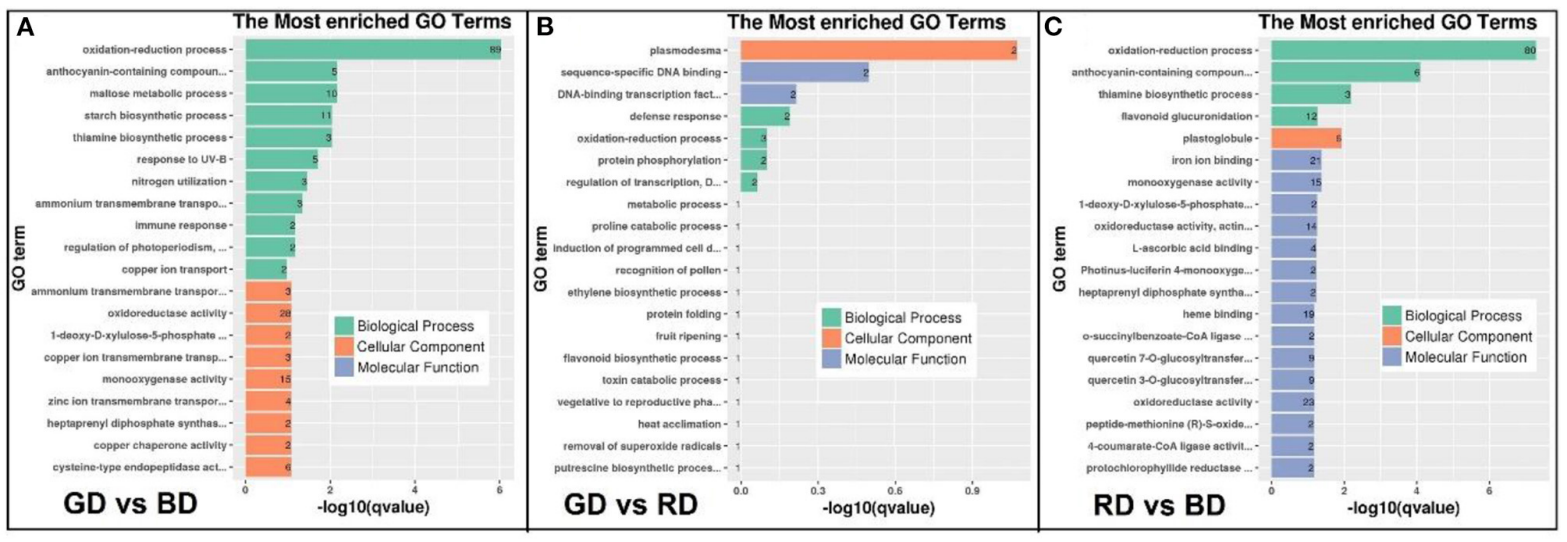
The GO enrichment analysis of DEGs in the comparisons of GD vs. BD **(A)**, GD vs RD **(B)**, and RD vs. BD **(C)** in three main categories. BP, biological process; CC, cellular component; MF, molecular function.

To reveal the function of green light-induced transcriptomes under drought stress, GO enrichment and KEGG pathway analysis were performed to categorize the DEGs. A total of 417, 10, and 386 DEG in the comparisons of GD vs. BD, GD vs. RD, and RD vs. BD were annotated into three major GO categories, respectively (Supplementary Table 2). In the comparison of GD vs. BD, the top 20 significantly enriched GO terms of DEGs were categorized into “biological process” and “cellular process” (Figure 5A). In the biological process category, the GO terms markedly enriched in the comparison of GD vs. BD included “oxidation-reduction process,” “anthocyanin-containing compound metabolic process,” and “maltose metabolic process,” and “starch biosynthetic process.” In the cellular process category, the significantly enriched GO terms were observed in “ammonium transmembrane transport,” “oxidoreductase activity,” and “1-deoxy-D-xylulose-5-phosphate” (Figure 5A). Compared with the other two comparisons, the enriched GO terms of the GD vs. RD comparison were not very complex. In the cellular component, “plasmodesma” was significantly enriched, while “sequence-specific DNA binding” and “DNA-binding transcription factors” were significantly enriched in the category of molecular function. Furthermore, the most significantly enriched GO terms of GD vs. RD comparison were identified in the processes of “defense response,” “oxidation-reduction process,” “protein phosphorylation,” and “regulation of transcription” (Figure 5B). With respect to the comparison of RD vs. BD, the significant enriched GO terms in the biological process included “oxidation-reduction process,” “anthocyanin-contained compounds,” “thiamine biosynthetic process,” and “flavonoid glucuronidation,” while the top five most significantly enriched GO terms in the molecular function category was identified in the processes of “iron ion binding,” “monooxygenase activity,” “1-deoxy-D-xylulose-5-phosphate,” “oxidoreductase activity,” and “L-ascorbic acid-binding.” In the category of the cellular compound, only “plastoglobule” was filtered into the top 20 significantly enriched GO terms in the RD vs. BD comparison (Figure 5C).

**Figure 6:**
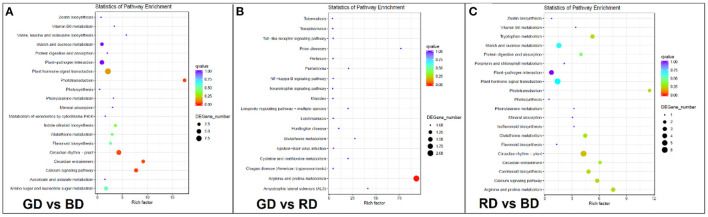
KEGG pathway enrichment analysis of the annotated DEGs in the comparisons of GD vs BD **(A)**, GD vs. RD **(B)**, and RD vs. BD **(C)**. The Y-axis indicates the KEGG pathway, while the X-axis presents the enrichment factor, which is the ratio of DEGs enriched to specific KEGG pathways to DEGs enriched to all KEGG pathways. The dot size and the dot color indicate the number of DEGs of the pathway and q value, respectively.

To further explore the biological functions of DEGs, the pathway enriched analysis based on the KEGG database was performed. When comparing each biological category, a higher number of DEGs were detected in the comparisons of GD vs. BD and RD vs. BD compared with GD vs. RD comparison, and the top 20 pathways in plants with the highest enrichment levels were listed in Figure 6. Among these KEGG pathways, DEGs in the comparison of GD vs. BD were mostly enriched in “phototransduction,” “circadian entrainment,” “calcium signaling pathway,” “circadian rhythm-plant,” and “plant hormone signal transduction” (Figure 6A). However, due to the less amount of DEGs detected in the comparison of GD vs. RD, the most significantly enriched pathway was detected in “arginine and proline metabolism” (Figure 6B). Furthermore, the top 5 pathways in enrichment degree were “phototransduction,” “arginine and proline metabolism,” “calcium signaling pathway,” “circadian entrainment,” and “circadian rhythm-plant” under RD vs. BD comparison (Figure 6C).

### Transcription Factors Identified by DEGs and PPI Network Analysis

The transcription factors among different comparisons were identified from DEGs and summarized in Supplementary Table 3. Among the DEGs of GD vs. BD comparison, 610 transcription factors were identified, including AP2/ERF (18), bHLH (16), bZIP (7), C2H2 (17), HSF (6), NAC (17), MYB/MYB-related (19), and WRKY (8). In the comparison of GD vs. RD, 13 transcription factor was identified, including AP2/ERF (1), C2H2 (1), WRKY (2), and SNF2 (1). Furthermore, there were 503 transcription factors detected in the comparison of RD vs. BD, including AP2/ERF (11), bHLH (15), bZIP (8), C2H2 (12), HSF (5), NAC (16), MYB/MYB-related (22), and WRKY (6).

**Figure 7:**
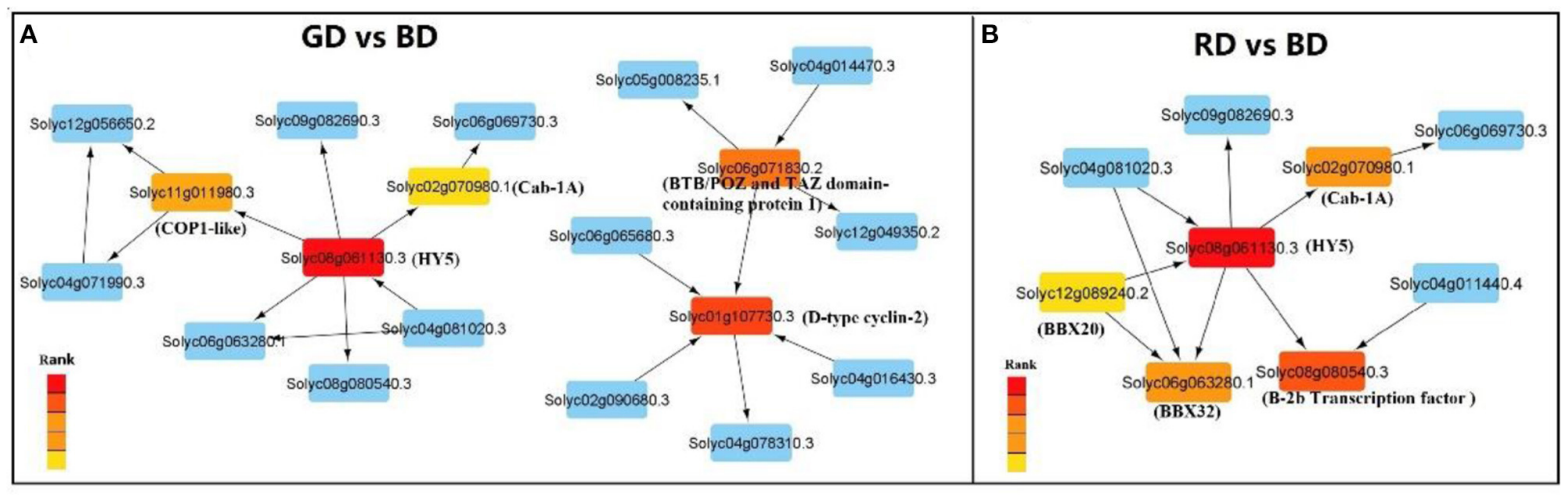
Protein-protein interaction (PPI) network analysis based on the DEDs in the comparisons of “GD vs. BD” **(A)** and “RD vs. BD” **(B)**. Nodes depict proteins encoded by related genes and PPI are represented by edges in the network; the top five hub DEDs encoded proteins are represented by various colors (red: high rank; Yellow: low rank) in the PPI networks.

To further investigate the hub genes involved in light spectral-induced drought tolerance, we first constructed the PPI network using screened DEG by Cytoscapeversion 3.8.0 (Figure 7). The top five hub proteins encoded by DEGs were further identified in the PPI network based on radiality analysis by employing cytohubba (Supplementary Tables 4, 5). In the comparison of GD vs. BD, two key hub proteins were identified in two sub-PPI networks. One of the key hub proteins-HY5 related gene (*Solyc08g061130.3*) was down-regulated, which directly acted with the other two hub protein: Solyc11g011980.3 (E3 ubiquitin-protein ligase COP1-like isoform X1) and Solyc02g070980.1 (chlorophyll a/b-binding protein Cab-1A). The other key hub gene-encoded protein was Solyc01g107730.3 (CycD3), which was directly affected by Solyc06g071830.2 (BTB/POZ and TAZ domain-containing protein 1) to participate in the regulation process of Solyc04g014470.3 (transcription factors, MYB20-like) (Figure 7A). In the comparison of RD vs. BD, Solyc08g061130.3 (transcription factors, HY5) was also identified as the key hub protein, directly interacting with other four hub proteins: Solyc08g080540.3 (heat stress transcription factor, B-2b), Solyc02g070980.1 (chlorophyll a/b-binding protein Cab-1A), Solyc06g063280.1 (B-box zinc finger protein 32, BBX32), and Solyc12g089240.2 (B-box zinc finger protein 20, BBX20) (Figure 7B). Furthermore, no key hub proteins were identified by PPI analysis in GD vs. RD comparison due to the few DEGs detected in this comparison (Supplementary Table 4). These results indicate that HY5 might play role pivotal role in the light spectral-induced regulation of plant drought stress.

### Differentially Expressed Genes Involved in ABA Metabolism and ABA Signal Transduction

**Figure 8:**
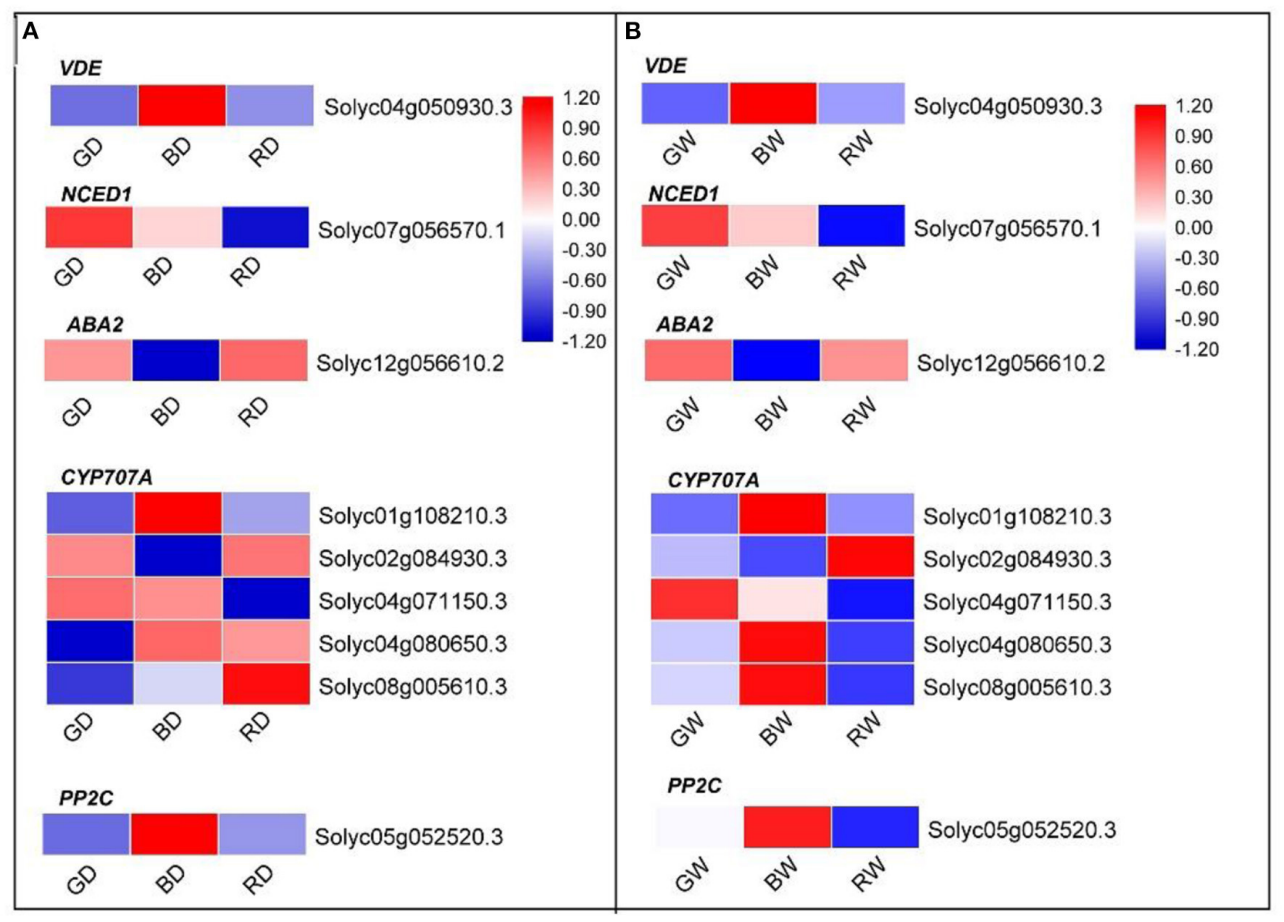
The heatmap diagram of relative expression profiles of DEGs involved in ABA synthesis and ABA signaling transduction in response to red, blue, and green light under drought conditions **(A)** and well-water conditions **(B)**. Gene expression is shown in a heatmap with color scale representing log_2_ (FPKM) (blue: low expression level; red: high expression level). A scale indicating the color assigned to log_2_ (FPKM) is shown to the right of the heatmap.

Regardless of water conditions, the expression pattern of plant hormone metabolism and signaling pathway-related DEGs under green light was significant to those under red and blue light. More DEGs were involved in ABA metabolism, signaling, and responses (Supplementary Figure 1). To further investigate how ABA-related genes responded to different light spectra, the expression of ABA metabolism- and ABA signaling transduction-related genes were filtered from the DEGs and listed in Figure 8. Eight differentially expressed genes involved in ABA biosynthesis and one ABA signaling transduction-related gene were detected. Regardless of water conditions, the transcription level of the violaxanthin de-epoxidase related gene (*Solyc04g050930.3, VDE*) was highest under blue light, followed by green light and red light. The highest and lowest expression level of *NCED1* (*Solyc07g056570.1*) encoding 9-cis-epoxycarotenoid dioxygenase (NCED) was found under green light and red light, respectively (Figures 8A,B). Compared with blue light, *ABA2* (*Solyc12g056610.2*), encoding another important enzyme of ABA biosynthesis, was up-regulated under green light and red light. The cytochrome P450 monooxygenase (P450) encoded by *CYP707As*, plays an important role in the catabolism of ABA. Five *CYP707As* were identified under different light treatments. Regardless of water condition, three CYP707A related genes (*Solyc01g108210.3, Solyc04g080650.3*, and *Solyc08g005610.3*) were down-regulated under green light when compared with those under blue (Figures 8A,B). Under the well-water condition, the expression levels of *Solyc01g108210.3, Solyc04g071150, Solyc04g080650.3*, and *Solyc08g005610.3* under red light were lower than those under blue light (Figure 8B); however, only two identified *CYP707As* (*Solyc01g108210.3* and *Solyc04g071150*) were down-regulated under RD compared with those under BD. In addition, the PP2C related gene (*Solyc05g052520.3*) of plant leaves under green and red light was down-regulated compared with that of blue light treated plants (Figures 8A,B). These results suggest that the ABA biosynthesis, metabolism, and ABA signaling transduction are regulated by light spectra. Compared with red, green light not only enhances ABA biosynthesis and concomitantly reduces ABA degradation but is also involved in ABA signaling transduction in tomato plants, while green light is more efficient in promoting ABA biosynthesis in tomato seedlings.

### Validation of Differently Expressed Genes by qPCR

The reliability of RNA-Seq data was verified by qRT-PCR using 10 randomly selected DEGs. The transcript profiles of these selected genes in the qRT-PCR analysis showed a similar pattern as being identified by the FPKM from RNA-seq under corresponding treatments (Supplementary Figure 2). These results confirm the reliability of RNA-Seq data.

## Discussion

Light is one of the most important environmental cues in the regulation of plant growth, development, and stress responses. A better understanding of the interaction between light and stress will provide useful information for helping both greenhouse and vertical farming production, with increasing crop production and concomitantly improving resource use efficiency. The present study aimed to explore the mechanism of green light on drought tolerance of tomato plants from a physiological and molecular perspective. For this purpose, this study investigated the effects of monochrome red, blue, and green light on photosynthetic performance, stomatal response, and endogenous ABA synthesis, and further characterize the differences in the transcriptome under drought stress.

In the present study, we found that compared with well-watered plants, the photosynthesis of red and blue light-treated plants was significantly decreased, while the photosynthetic capacity under green light showed a slight decline during 9 days of drought treatments (Figure 1). Together with comparable biomass of plants exposed to different light spectra under drought stress (Table 1) and the low electrolyte leakage under green light (Figure 3A), these results confirm the positive function of green light on alleviating drought stress-induced decreases in plant growth. This is consistent with our previous studies that green light showed positive effects on maintaining photosynthetic capacity and enhancing the stress tolerance of plants under continuous light and drought conditions (Bian et al., 2018, 2019).

Stomata are the gateway for CO_2_ uptake and water loss from plant leaves by transpiration (Osakabe et al., 2014). Apart from being regulated by internal cues (e.g., phytohormones and Ca${}_{2}^{+}$ signal), the stomatal aperture is controlled by external light spectra. In the present study, the g_s_, Tr, and stomatal aperture of well-watered plants were lower under green light than those under red and blue light (Figure 2). These findings may lie in the fact that red and blue light facility stomata opening, while green light less efficient in promoting stomatal opening and strong green light suppressed the blue-light dependent stomatal opening (Frechilla et al., 2000; Shimazaki et al., 2007). However, the effect of light spectra on g_s_ was not in line with the changes of Tr and stomatal aperture under drought stress (Figure 2). In plants, transpiration is not only regulated by light-regulated stomatal aperture but also affected by other environmental factors, including soil and atmospheric moisture stresses (Durand et al., 2019). Furthermore, the g_s_ depends on the aperture, size, and density of stomata in plants (Monda et al., 2016). Thus, g_s_ of plants is not always correlated with Tr and stomatal aperture, especially under different abiotic stress conditions (Durand et al., 2019; Zhang et al., 2020).

Compared with RD and BD, the relatively higher instantaneous WUE and RWE and concomitantly lower electrolyte leakage and stomatal aperture under GD were further demonstrated our previous report that green light positively enhanced tomato plant drought stress tolerance via stomatal regulation (Bian et al., 2019). As an important phytohormone, ABA plays a vital role in plant growth, development, and stress tolerance, such as stomatal regulation, cold, and drought tolerance (Chen et al., 2020). In this study, the relatively high ABA content in green light treated plants (Figure 2E) suggests that ABA may be involved in the green light-induced regulation of stomata and drought stress response. To reveal the underlying mechanism of green light in the regulation of drought tolerance, RNA-Seq analysis was employed to uncover the effects of light spectra on the global expression profiling of plants under drought stress. According to the basis of KEGG enrichment analysis, it was confirmed that “light transduction” and “plant hormone signal transduction” were involved in spectral regulation under drought stress (Figure 6). The related genes encoded the key enzymes of ABA synthesis, metabolism, and signaling were successfully filtered from the DEGs (Figure 8). Thompson et al. (2007) reported that overexpression of *LeNCED1* greatly reduced stomatal aperture through enhancing ABA synthesis in plant leaves. Furthermore, the ABA content can be mirrored by the expression of *ABA2*, another important gene in the ABA synthesis pathway (Tripathi et al., 2016). Therefore, regardless of water condition, the high ABA content and low stomatal aperture in green light-treated plants in the present study might be attributed to the up-regulated *NCED1* (*Solyc07g056570.1*) and *ABA2* (*Solyc12g056610.2*) compared with that under red and blue light, respectively (Figure 8).

The ABA content in plants depends on the balance between its biosynthesis and catabolism. Up-regulated *VDE* gene expression decreases ABA synthesis (Pastori et al., 2003). A cytochrome P450 monooxygenase (P450) encoded by *CYP707As* is a key enzyme in ABA catabolism (Kushiro et al., 2004). Our present study showed that *VDE* and most *CYP707As* identified from DEGs were up-regulated under blue, while most *CYP707As* under green light were down-regulated when compared with transcription levels under red and blue light under drought conditions (Figure 8). These results indicate that the relatively high ABA content under green light was attributed to green light-induced ABA synthesis and concomitantly a decrease of ABA catabolism.

**Figure 9:**
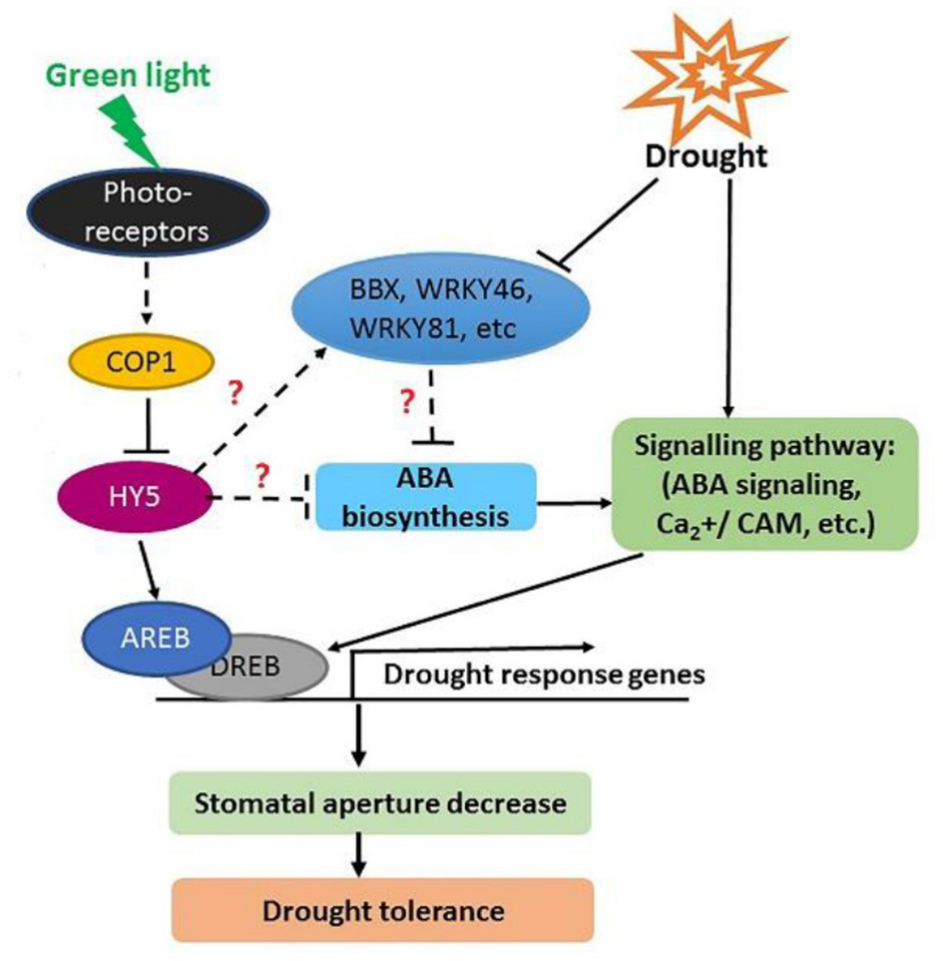
A proposed model of green light enhances tomato drought tolerance.

ABA signal transduction plays a core role in ABA-dependent responses to abiotic stress. The signal transduction module of ABA is made up of three protein classes: Pyracbactin Resistance/Pyracbactin resistance-like/Regulatory Component of ABA Receptor (PYR/PYL/RCARs), Protein Phosphatase 2Cs (PP2Cs), and SNF1-related protein kinase 2s (SnRKs) (Danquah et al., 2014). PYR/PYL/RCARs are proved to be the ABA receptors, while PP2Cs and SnRKs act as the negative and positive regulators in ABA signaling, respectively (Park et al., 2009; Umezawa et al., 2010). The PP2C activity is inhibited by the PYR/PYL/RCAR-PP2C complex formation (Park et al., 2009; Santiago et al., 2009). The inhibition of PP2Cs activity allows SnRKs to actively target membrane proteins, ion channels, and transcription factors, and facilitate transcription of ABA-responsive genes, thereby regulating plant growth, development, and stress responses (Umezawa et al., 2010; Soon et al., 2012). Previous studies demonstrate that SnRK2.6 (open stomata, OST1) regulates fast ABA responses resulting in stomatal closure, which is inhibited by PP2Cs via dephosphorylation of serine 175 (Umezawa et al., 2009; Vlad et al., 2009). In our present study, the significantly different transcription levels of one PP2C related gene (*Solyc05g052520.3*) under different light spectra suggest light spectra play important roles in ABA signal transduction. Together with the lower stomatal aperture (Figure 2D) and drought-induced damage under green light (Figure 3), the green-light induced drought tolerance in this present study may partly attribute to ABA signal transduction-induced the fast stomatal closure (Figure 9).

Transcription factors play pivotal roles in plant tolerance to various stresses through triggering or retarding downstream gene expression. Several stress-related transcription factors of bZIP, MYB/MYB-related, and WRKY were involved in the light spectral regulation under drought (Supplementary Table 3). According to the PPI network analysis, a bZIP family transcription factor, HY5, was identified as the hub gene in the regulation of light spectral on plant drought stress responses (Figure 7). In plants, HY5 integrates light signal and endogenous ABA to regulate plant development and stress tolerance (Xu et al., 2014). In addition, HY5 acts downstream of COP1 to suppresses ABA-regulated inhibition of seedling development (Yadukrishnan et al., 2020). Compared to blue light, green light led to down-regulation of HY5 related gene (Supplementary Table 3) but promoted ABA synthesis and signal transduction related gene expression (Figure 8), indicating that HY5 might act as a negative regulator in the green light-induced drought responses via modulating ABA synthesis and related signal transduction. To reveal this hypothesis, more detailed ongoing studies are still needed. Furthermore, WRKYs is another important transcription factor family and plays a significant role in protecting plants against drought stress. Activated expression of *AtWRKY57* improves drought tolerance by elevation of ABA levels in *Arabidopsis* (Jiang et al., 2012). Jaffar et al. (2016) showed that overexpression of *CmWRKY10* in transgenic chrysanthemum plants improved tolerance to drought stress via up-regulating NCED related gene expression. Although no PPI network was constructed in the comparison of GD vs. RD because of few DEGs identified in this comparison, two important WRKY transcription factors, WRKY46 and WRKY81, were filtered and their related gene expressions were down-regulated by green light under drought conditions (Supplementary Table 3). Previous studies demonstrated that WRKY46 and WRKY81 were negative regulators for plant drought tolerance (Chen et al., 2017; Ahammed et al., 2020b), and their inhibition of drought tolerance is involved in the ABA-mediated pathway and the light-dependent stomatal opening in guard cells (Ding et al., 2014; Ahammed et al., 2020a). Compared with red light-treated plants, the down-regulated *WRKY46*, and *WRKY81* and relatively high ABA content with the up-regulated NCED related gene under green light suggest that *WRKY46* and *WRKY81* might indirectly be involved in the green light-induced drought tolerance via ABA-dependent pathway.

To date, the green light receptors have not been confirmed, but some studies indicate that green light might be involved in the regulation of plant growth and morphogenesis through the mediation of blue light receptors such as phototropin and cryptochrome (Bouly et al., 2007; Matthews et al., 2020). Based on the results of the present transcriptomic analysis and previous studies, a putative regulatory network of green-induced drought tolerance of tomato seedlings was proposed (Figure 9). Under green light radiation, the green light receptor perceives light signals and interacts with COP1, which directly regulates HY5. HY5 could interact with other transcription factors, like AREBs, DREBs, or WRKYs, to regulate the transcription of downstream genes involved in drought stress tolerance (He et al., 2018). Furthermore, green light promotes ABA accumulation via triggering the expression of ABA biosynthesis-related genes, such as *NCED*1 and *ABA2*, and meanwhile down-regulating the expression of ABA degradation-related gene-*CYP707A*. The resulting accumulated ABA induces signaling pathways, such as ABA signaling and Ca^2+^/CaM, to enhance drought tolerance. Further studies at physiological and molecular levels should be conducted to provide more precise insight into the mechanisms of green light-induced drought tolerance. Successful identification of these key genes would give a great opportunity for breeding new varieties with high resources use efficiency or/and stress tolerance that are suitable to vertical farming.

## Conclusion

The present study confirmed that green light has a positive function in alleviating the detrimental effects of drought stress on plant growth and photosynthetic capacity via stomatal aperture regulation. According to the analysis of the transcriptomic dataset, the identified those key genes encoding ABA synthesis and signaling revealed the involvement of the ABA-dependent pathway in green light-induced drought tolerance via stomatal regulation. The responses of transcription factors, including HY5, WRKY46, and WRKY81, to green light-induced drought responses, were also identified. The transcription data lay the groundwork for further revealing the mechanism of green light-induced stomatal movement and stress tolerance under drought conditions. However, further studies are needed to decipher the regulatory mechanisms of HY5, WRKY46, and WRKY81 in green light-induced drought tolerance. Furthermore, it is important to explore how genetic variation and phenotypic plasticity respond to abiotic stresses with allowing specific traits to be presented. We expect to be able to create an LED lighting system that can be programmed to generate an optimized wavelength recipe unique for each plant species.

## Data Availability Statement

The original contributions presented in the study are publicly available. This data can be found here: NCBI SRA database BioProject accession number PRJNA691997 (https://www.ncbi.nlm.nih.gov/bioproject/PRJNA691997).

## Author Contributions

ZB, CL, and QY conceived the original research plan and designed the experiment. ZB, YW, XZ, SG, and KH performed the experiments and analyzed the data. ZB and YW wrote the manuscript, while ZB and CL reviewed and edited the manuscript.

## Funding

This research was financially funded by Institute of Urban Agriculture Internal Funding (S2021001), the Technology Innovation Program of the Chinese Academy of Agricultural Sciences (34-IUA-03), Nottingham Trent University Q&R Funding (01 ARE RA926), Science and the National Natural Science Foundation of China (No. 51708283), and the Natural Science Foundation of Jiangsu Province (No. BK20171011).

## Conflict of Interest

The authors declare that the research was conducted in the absence of any commercial or financial relationships that could be construed as a potential conflict of interest.

## Publisher's Note

All claims expressed in this article are solely those of the authors and do not necessarily represent those of their affiliated organizations, or those of the publisher, the editors and the reviewers. Any product that may be evaluated in this article, or claim that may be made by its manufacturer, is not guaranteed or endorsed by the publisher.

## Supplementary Material

Supplementary Figure 1 The Hierarchical clustering of plant hormone related DEGs of tomato treated with different light spectra and water conditions. Fragments per kilobase of transcript per million fragments mapped (FPKM), and relative expression of DEGs between different light and water treatments were calculated as log2 (FPKM) of differentially expressed genes, log_2_ (FPKM) of differentially expressed genes were calculated. A scale indicating the color assigned to log_2_ (FPKM) is shown to the right of the cluster.

Supplementary Figure 2 The expression patterns of 10 randomly selected DEGs by RNA-Seq and qPCR. Gene expression is shown in a heatmap with color scale representing log_2_ (fold change) (blue: low expression level; red: high expression level). A scale indicating the color assigned to log_2_ (fold change) is shown to the right of the heatmaps.

Supplementary Table 1 The details of the primers used for RNA-Seq data validation by qPCR.

Supplementary Table 2 GO enrichment analysis.

Supplementary Table 3 The transcription factor analysis of plants under drought stress by DEGs.

Supplementary Table 4 Protein-protein interaction (PPI) network analysis based on the DEDs of the comparsion GD and BD.

Supplementary Table 5 Protein-protein interaction (PPI) network analysis based on the DEDs of the comparsion RD and BD.
