# Supplementary figures and images for: A Transcriptome Analysis Revealing the New Insight of Green Light on Tomato Plant Growth and Drought Stress Tolerance
Source: Front Plant Sci. 2021 Oct 21;12:649283. doi: 10.3389/fpls.2021.649283 (PMC8566944; doi:10.3389/fpls.2021.649283)

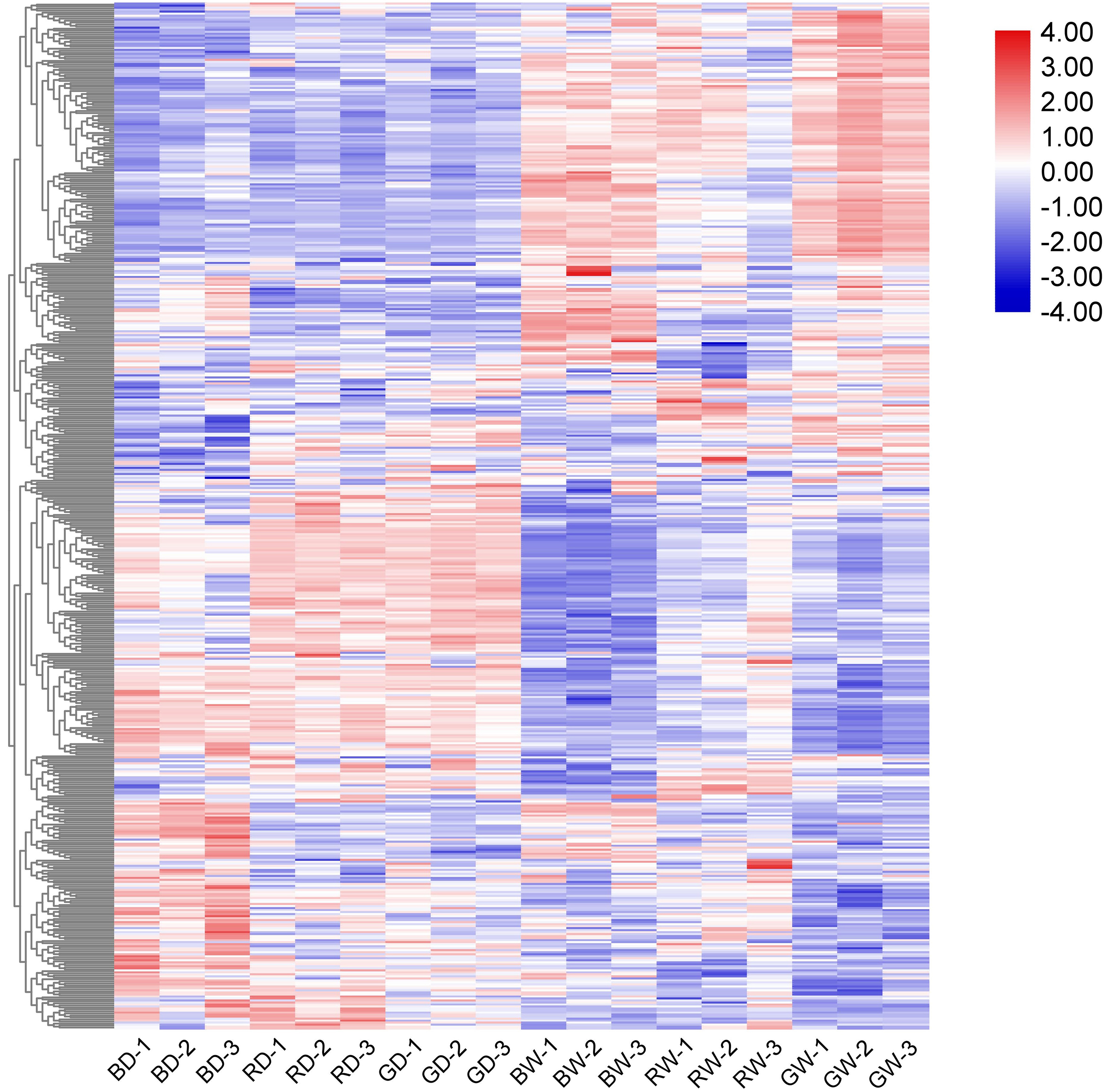

Supplement: Supplementary Figure 1 — The Hierarchical clustering of plant hormone related DEGs of tomato treated with different light spectra and water conditions. Fragments per kilobase of transcript per million fragments mapped (FPKM), and relative expression of DEGs between different light and water treatments were calculated as log2 (FPKM) of differentially expressed genes, log2 (FPKM) of differentially expressed genes were calculated. A scale indicating the color assigned to log2 (FPKM) is shown to the right of the cluster. [file Image_1.JPEG]

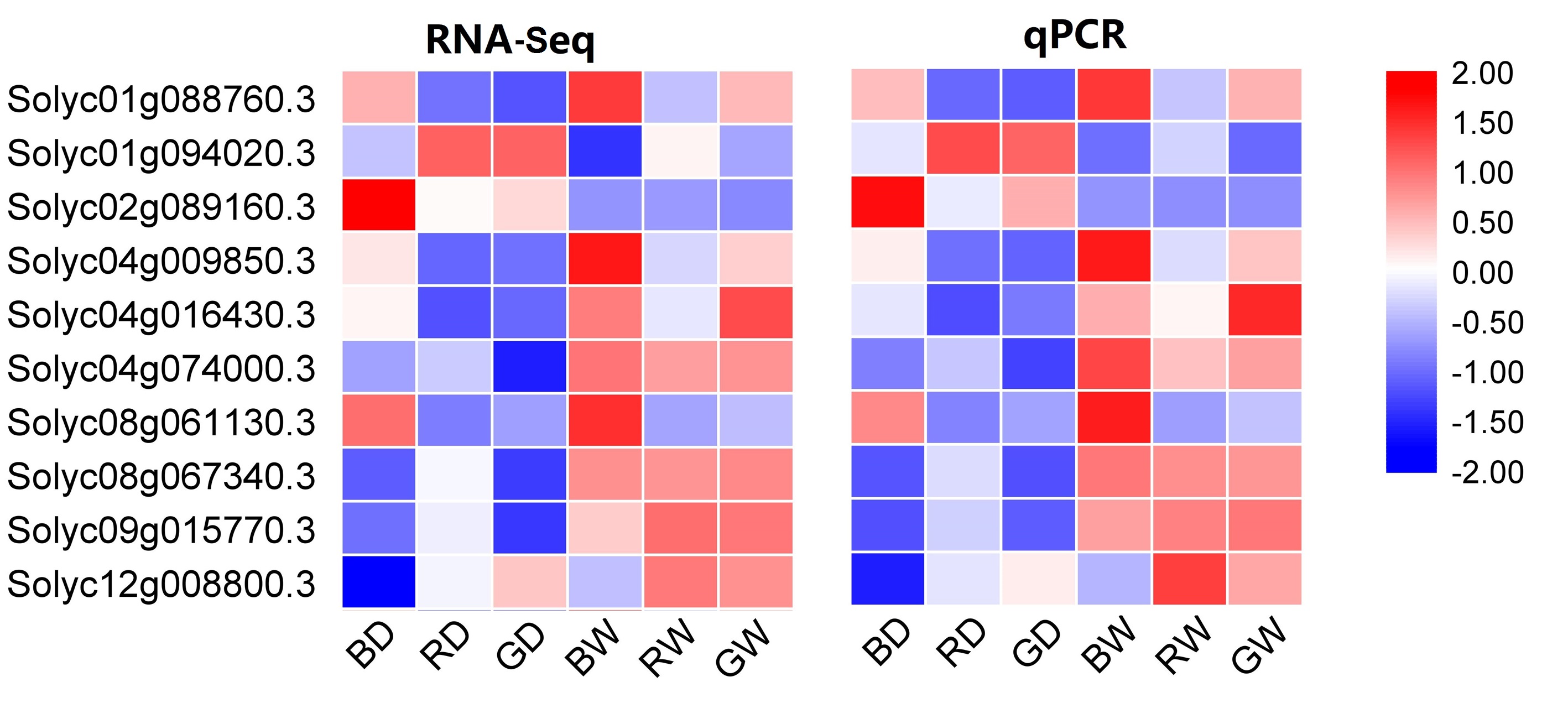

Supplement: Supplementary Figure 2 — The expression patterns of 10 randomly selected DEGs by RNA-Seq and qPCR. Gene expression is shown in a heatmap with color scale representing log2 (fold change) (blue: low expression level; red: high expression level). A scale indicating the color assigned to log2 (fold change) is shown to the right of the heatmaps. [file Image_2.JPEG]
