# Supplementary material for: A Transcriptome Analysis Revealing the New Insight of Green Light on Tomato Plant Growth and Drought Stress Tolerance
Source: Front Plant Sci. 2021 Oct 21;12:649283. doi: 10.3389/fpls.2021.649283 (PMC8566944; doi:10.3389/fpls.2021.649283)
Supplement: Supplementary Table 1 — The details of the primers used for RNA-Seq data validation by qPCR. [file Table_1.DOCX]

| Table S1. The details of the primers used for RNA-Seq data validation by qPCR | | |
| --- | --- | --- |
| Access No. | Gene Name | Primer sequences (5'-3') |
| Solyc01g088760.3 | CYP7142C | TGGACCTTACTGGGCTCATC |
|  |  | CGTCCACCTTAACCTCCGAT |
| Solyc01g094020.3 | At2g45590 | ATGTGGATCAGTTGCCGGAG |
|  |  | CCACACTAACCCCGTCGAAA |
| Solyc02g089160.3 | CYP85A1 | AGCCTGGAACACCAAAACTCA |
|  |  | TGGTGCTTCAACTCTTGGGA |
| Solyc04g009850.3 | BX6 | ACCTTGTCCTGAGCCCAATAA |
|  |  | AGCTGCATAAGGTCACCAAGA |
| Solyc04g016430.3 | CKX5 | TCGAATGCCGGAATCAGTGG |
|  |  | ACCAAACTGGCCTAGTCCTC |
| Solyc04g074000.3 | MDIS1 | CACTCAATGGCGAAGGGACA |
|  |  | TCCCGTCTTTCAACGTCTCC |
| Solyc08g061130.3 | HY5 | ATCTGGAAGCAAGGGTGAAGG |
|  |  | GCACCTGCTGTTGTGTTCTTC |
| Solyc08g067340.3 | WRKY46 | ACAATCATCCCCAACCACCTC |
|  |  | CTTCGACAACGAAGACGCCA |
| Solyc09g015770.3 | WRKY81 | ATTCTGATGTCGTCGTCGCT |
|  |  | CAAGCATGACCATCATCCACC |
| Solyc12g008800.3 | MYBS3 | CAAGGACTCCAACACAAGTTGC |
|  |  | TTTGTTGTTGCTCCTTGCCAT |
